# Phalloplasty Complicated by Penile Artery Thrombosis, Recurrent Extended-Spectrum Beta-Lactamase (ESBL) Urinary Tract Infection (UTI), Colovesical Fistula, and Enterococcus Faecalis Endocarditis

**DOI:** 10.7759/cureus.19716

**Published:** 2021-11-18

**Authors:** Zachary Gilbert, JP Markovic, David Stultz

**Affiliations:** 1 Internal Medicine, Kettering Medical Center, Kettering, USA; 2 Cardiovascular, Kettering Medical Center, Kettering, USA

**Keywords:** phalloplasty, penile artery thrombosis, esbl uti, colovesical fistula, mitral valve endocarditis

## Abstract

*Enterococcus faecalis* is an enteric microorganism that, if introduced into the vasculature, is an uncommon cause of infective endocarditis. Timely diagnosis, appropriate treatment measures, and close follow-up are key to therapeutic success. Antibiotic therapy is the mainstay of therapy, and surgical intervention is sometimes indicated. Here we present a novel case of a 45-year-old transgender male with Factor V Leiden deficiency who was found to have *Enterococcus faecalis *mitral valve endocarditis due to the postoperative complications of colovesical fistula formation leading to extended-spectrum beta-lactamase (ESBL) urinary tract infection (UTI) and *E. faecalis* bacteremia in the setting of recent phalloplasty, mastectomy, and vaginal eversion.

## Introduction

Although mitral valve endocarditis with severe mitral valve regurgitation is not rare, we present a unique set of events not reported in the literature before involving infective endocarditis and gender reassignment surgery. While *Enterococcus faecalis* is a less common cause of infective endocarditis, *Staphylococcus aureus* is the leading culprit. Infective endocarditis should be suspected with fever, certain cardiac risk factors (prior endocarditis, valvular or congenital heart disease, prosthetic heart valve, or cardiac device), and other noncardiac factors (immunosuppression, IV drug use, indwelling lines, recent dental or surgical procedure). A lapse in timely diagnosis and appropriate treatment can lead to complications such as valvular insufficiency, new-onset heart failure, embolic phenomena, and sepsis. Swift diagnosis of infectious endocarditis, appropriate intervention, and proper antibiotic coverage is key to overall patient prognosis.

## Case presentation

A 45-year-old transgender male with a history of Guillain Barre Syndrome and heterozygous Factor V Leiden underwent gender reassignment surgery, including phalloplasty, mastectomy, and vaginal eversion. The phalloplasty was complicated by postoperative penile artery thrombosis, recurrent episodes of extended-spectrum beta-lactamase (ESBL) *Klebsiella* urinary tract infection (UTI) from a chronic suprapubic catheterization, and colovesical fistula.

**Figure 1:**
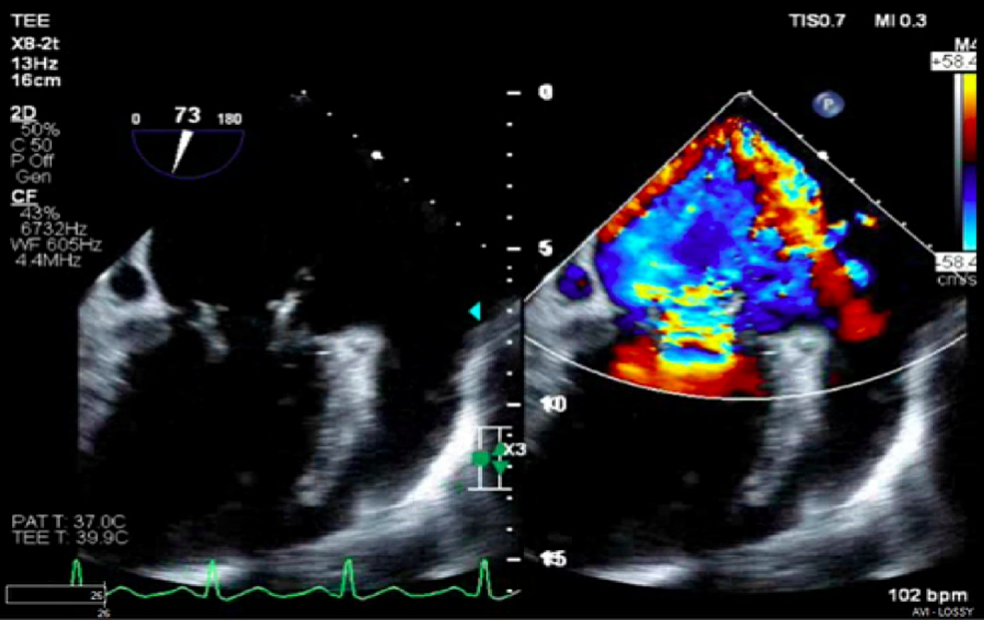
2.1 cm mitral valve leaflet mobile vegetation viewed on transesophageal echocardiogram (TEE)

**Figure 2:**
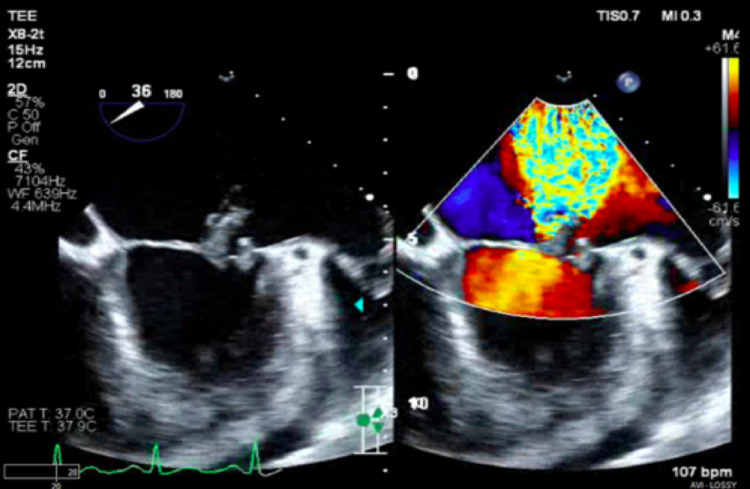
Transesophageal echocardiogram (TEE) image depicting moderate-to-severe mitral regurgitation with the jet bouncing off the back wall of the left atrium

The patient presented with lethargy, shortness of breath, 15 lb weight gain, and lower extremity edema for the past three months. Physical exam revealed tachycardia, holosystolic murmur at the apex, faint bibasilar crackles, and right lower extremity edema. Laboratory work showed leukocytosis (WBC 11.6 K/uL), creatinine 0.7 mg/dL, hemoglobin 8.8 g/dL, B-type natriuretic peptide of 610 pg/mL, and D-dimer of 3469 ng/mL. CT chest revealed cardiomegaly, pulmonary edema, bilateral lower lobe consolidations, and pleural effusions, a large pericardial effusion, and a 5x10 cm perisplenic abscess found to be secondary to septic emboli. Echocardiogram showed a 2.1 cm mobile vegetation on the anterior leaflet of the mitral valve (Figure 1), moderate-severe mitral valve insufficiency (Figure 2), ejection fraction of 65%, markedly dilated left atrium, elevated peak pulmonary artery pressure at 57 mmHg, and large pericardial effusion without evidence of tamponade. Broad-spectrum IV antibiotics were started for infective endocarditis and diuresis for new-onset heart failure. Splenic fluid and blood cultures grew *Enterococcus faecalis*. He underwent subsequent cardiothoracic surgery with an On-X mechanical mitral valve (On-X Life Technologies Inc., Austin, TX). Unfortunately, the postoperative course was complicated by mediastinal thrombus formation and hemothorax requiring surgical exploration. Once stabilized, the patient was discharged and upon discharge was placed in IV penicillin G and ceftriaxone to complete six weeks of antibiotic therapy.

## Discussion

Infective endocarditis is described as a microbial infection of the endocardium manifested as a lesion made up of platelets, fibrin, microbes, and inflammatory cells. Risk factors for the development of infective endocarditis include a history of prior infective endocarditis, the presence of a prosthetic heart valve or cardiac device, structural or congenital heart disease, intravenous drug use, and a recent invasive procedure. The most common cardiovascular factor predisposing patients to infective endocarditis is mitral valve prolapse, but in the developing world, this is due to rheumatic heart disease. The most common microbial culprits, in order of frequency, are methicillin-susceptible *Staphylococcus aureus*, viridans group streptococci, coagulase-negative staphylococci, enterococci, *Streptococcus gallolyticus*, other streptococci, fungi, gram-negative HACEK (Haemophilus species, Aggregatibacter species, Cardiobacterium hominis, Eikenella corrodens, and Kingella species) bacilli, and gram-negative non-HACEK bacilli [1]. Despite low prevalence, polymicrobial infections are the most common cause in patients with intravenous drug use [2]. Presentation varies tremendously from an acute and rapidly progressing infection to a chronic infective process [3]. Clinicians should suspect endocarditis on their differential diagnosis with any patient that presents with fevers, night sweats, and signs of systemic illness in high-risk patients as described above. Duke criteria is a widely accepted tool to aid in the diagnosis of infective endocarditis, which is summarized in Table 1.

**Table 1:** Duke criteria aiding in the diagnosis of infective endocarditis HACEK: Haemophilus species, Aggregatibacter species, Cardiobacterium hominis, Eikenella corrodens, and Kingella species

Major Criteria	Minor Criteria
1. Two separate positive blood cultures with typical endocarditis organisms (Staphylococcus aureus, Streptococcus viridans, Staphylococcus epidermidis, HACEK group)	1. Risk factor: IVDA or predisposing heart condition
	2. Fever >38℃
2. Evidence of echocardiographic involvement (vegetation, abscess, new dehiscence of prosthetic valve)	3. Immunological phenomena: glomerulonephritis, Osler nodes, Roth spots
	4. Embolic phenomena: pulmonary infarct, arterial emboli, Janeway lesion, conjunctival hemorrhage
	5. Positive blood culture that does not meet major criteria adjacent

*Enterococcus faecalis* is derived from the French word ‘entérocoque,’ which references its enteric bacterial origin, and ‘faecalis,’ which points toward its presence in feces. *Enterococcus faecalis* has been found to cause 5%-15% of community-acquired endocarditis and up to 30% of nosocomial-acquired endocarditis. Additionally, it causes roughly 15%-20% of urinary tract infections [4-5]. *Enterococcus faecalis* endocarditis has been reported to have a 26% prevalence in patients with Enterococcus faecalis bacteremia [6].

According to recommendations from the American Heart Association, enterococcus endocarditis should be treated with four to six weeks of intravenous penicillin or ampicillin in addition to aminoglycoside [7]. Treatment of *Enterococcus faecalis *endocarditis can be difficult given high levels of resistance to aminoglycosides, as well as nephrotoxicity. A combination of ampicillin plus ceftriaxone has been found to be an effective alternative [8-9]. Strict antibiotic adherence and close outpatient follow-up are important in overall treatment outcomes. Interestingly, a retrospective study found that half of the cases with *Enterococcus faecalis* without clear source identification were found to have colorectal neoplasms [10]. Thus if no clear source of infection is suspected, an outpatient colonoscopy should be obtained for possible source identification.

## Conclusions

*Enterococcus faecalis* is a less common cause of infective endocarditis. Timely diagnosis, appropriate treatment, and close follow-up are key to therapeutic success and avoidance of complications that include valvular insufficiency, new-onset heart failure, embolic phenomena, sepsis, and death. We present a rare sequence of events initiated by gender reassignment surgery complicated by postoperative penile artery thrombosis due to Factor V Leiden deficiency, which required a chronic suprapubic catheter. Recurrent ESBL urinary tract infections combined with chronic catheterization led to colovesical fistula formation and ultimately led to *Enterococcus faecalis* bacteremia and mitral valve infective endocarditis. Although a rare complication and set of events, it is our recommendation that more research and a greater understanding of the possible surgical complications of gender reassignment be made to improve patient outcomes.
